# The Efficacy of Transcutaneous Electrical Acupoint Stimulation as an Adjunct to Standard Bladder Training for Bladder Emptying After Radical Hysterectomy for Cervical Cancer: A Randomized Controlled Trial

**DOI:** 10.3390/curroncol33070424

**Published:** 2026-07-16

**Authors:** Ting Xu, Junya Ke, Yalin Yue, Zhiling Zhu, Mingzhi Zhao, Yun Wang

**Affiliations:** Department of Integrated Traditional Chinese and Western Medicine, Obstetrics and Gynecology Hospital of Fudan University, Shanghai 200090, China; xuting7685@fckyy.org.cn (T.X.); kejunya7833@fckyy.org.cn (J.K.); yueyalin7707@fckyy.org.cn (Y.Y.); zhuzhiling1023@fckyy.org.cn (Z.Z.)

**Keywords:** transcutaneous electrical acupoint stimulation, urinary retention, radical hysterectomy, cervical cancer, quality of life, randomized controlled trial

## Abstract

After radical hysterectomy for cervical cancer, postoperative bladder dysfunction is common. This randomized controlled trial evaluated whether adjunctive transcutaneous electrical acupoint stimulation (TEAS) reduces urinary retention. A total of 108 patients were assigned to a control group (standard bladder training, *n* = 53) or a TEAS group (standard training plus daily 30 min TEAS for 7 days from postoperative day 3, *n* = 55). The primary outcome was urinary retention (post-void residual > 100 mL on day 11). Secondary outcomes included residual volume, recovery time, recatheterization, urinary tract infection (UTI), treatment efficacy, and quality of life (SF-36). Urinary retention occurred in 35.8% of controls versus 23.6% of TEAS patients (*p* = 0.164). TEAS significantly reduced median residual volume (70 vs. 85 mL, *p* = 0.004), shortened median recovery time (2 vs. 4 h, *p* < 0.001), and lowered recatheterization (16.4% vs. 34.0%, *p* = 0.036). UTI rates were similar. Treatment efficacy favored TEAS (*p* = 0.009), and all eight SF-36 domains significantly improved (all *p* < 0.01). In conclusion, adjunctive TEAS did not significantly reduce the primary outcome of urinary retention but significantly improved multiple secondary bladder-emptying measures and quality of life after radical hysterectomy for cervical cancer.

## Simple Summary

After radical hysterectomy for cervical cancer, many women experience difficulty emptying their bladder, which often requires a urinary catheter for longer periods and lowers their quality of life. This study tested whether transcutaneous electrical acupoint stimulation—a non-invasive treatment that delivers mild electrical currents to specific body points—could improve bladder recovery in 108 patients. The treatment did not significantly reduce the main measure of urinary retention, but it did lower the need for re-insertion of the catheter, reduce leftover urine in the bladder, shorten the time to normal urination, and improve quality of life. These findings suggest that this safe, simple technique could be a valuable addition to standard care and should be tested further in larger studies.

## 1. Introduction

Radical hysterectomy with pelvic lymphadenectomy is the standard surgical treatment for early-stage cervical cancer. However, the extensive parametrial resection required for oncological control inevitably risks injury to the pelvic autonomic nerve plexus, leading to postoperative bladder dysfunction in a substantial proportion of patients [1]. Urinary retention is the most common manifestation, reported in 20–68% of cases, resulting in prolonged catheterization, increased risk of urinary tract infections (UTIs), and significant impairment of quality of life [2,3,4].

Current postoperative management relies primarily on indwelling catheterization and passive bladder training. These supportive strategies allow time for spontaneous nerve recovery but do not actively promote neurological regeneration or reliably prevent urinary retention. Geller [5] reviewed postoperative urinary retention management after urogynecologic surgery and highlighted the need for multimodal approaches. Bosch et al. [6] questioned whether routine screening for lower urinary tract dysfunction after major pelvic surgery is warranted, pointing to the lack of evidence-based protocols for postoperative bladder care. Heliövaara-Peippo et al. [7] reported in a 10-year follow-up study that lower urinary tract symptoms persist in a substantial proportion of patients after hysterectomy, suggesting that passive management strategies may be insufficient for long-term recovery. Hinman [8] emphasized that postoperative bladder overdistension is a preventable complication, yet effective preventive interventions remain limited. Harmacological options, including cholinergic agents and alpha-blockers, have shown limited efficacy and are often associated with adverse effects such as gastrointestinal disturbance and hypotension [5]. Therefore, safe, effective, and well-tolerated adjunctive interventions to accelerate bladder rehabilitation are urgently needed.

Transcutaneous electrical acupoint stimulation (TEAS) is a non-invasive technique that applies low-frequency electrical currents to traditional acupuncture points. TEAS is postulated to modulate the sacral micturition center (S2–S4) and improve local pelvic circulation, potentially facilitating the recovery of detrusor and sphincter coordination [9,10,11]. Preliminary studies have suggested potential benefits of electrical stimulation for voiding dysfunction [12,13]. However, the optimal stimulation parameters (e.g., frequency, intensity, duration) remain debated, and the mechanisms by which TEAS improves bladder function are not fully understood. Moreover, robust evidence from high-quality randomized controlled trials (RCTs) specifically evaluating TEAS for bladder recovery after radical hysterectomy is lacking.

To address this gap, we designed this RCT with the following objectives: to evaluate whether adjunctive TEAS reduces the incidence of urinary retention after radical hysterectomy for cervical cancer, and to assess its effects on secondary bladder-emptying measures, treatment efficacy, and quality of life. We therefore conducted this RCT to test the hypothesis that adjunctive TEAS, compared with standard bladder training alone, reduces the incidence of urinary retention and improves secondary bladder-emptying measures and quality of life in patients undergoing radical hysterectomy for cervical cancer. The principal findings indicate that although TEAS did not significantly reduce the primary outcome of urinary retention, it significantly improved several clinically important secondary outcomes, including recatheterization rate, residual urine volume, recovery time, and quality of life. These results provide evidence-based support for integrating TEAS into postoperative rehabilitation protocols.

## 2. Materials and Methods

### 2.1. Study Design and Participants

This exploratory randomized controlled trial adopted a prospective, single-center, open-label design. It was carried out at the Obstetrics and Gynecology Hospital of Fudan University in Shanghai, China, covering the period from February 2024 to December 2025. The study obtained trial registration on 31 January 2024 (ChiCTR2400080541). This study is part of an ongoing four-arm trial. According to the original protocol, the four arms include standard bladder training alone (control), standard bladder training plus TEAS, standard bladder training plus tamsulosin, and standard bladder training plus TEAS and tamsulosin, with a planned sample size of 50 patients per arm. The present manuscript reports the completed comparison between the control and TEAS groups, which completed enrollment and 30-day follow-up ahead of the other two arms. The full four-arm results will be reported separately once all arms have reached the required sample size and follow-up is completed. Patients were eligible if they met the following criteria: (1) female sex, aged 35 to 60 years, with a pathological diagnosis of cervical cancer (FIGO 2018 stages IB1-IIA2) and scheduled for radical hysterectomy; (2) surgical documentation confirming radical hysterectomy; (3) life expectancy of at least 12 months; and (4) provision of written informed consent. The age restriction was chosen to reflect the typical patient profile at our institution while reducing potential confounders, such as hormonal fluctuations in younger women and comorbidities in older patients, thereby enhancing cohort homogeneity for assessing TEAS efficacy. Exclusion criteria comprised: (1) prior pelvic irradiation; (2) age outside the 35–60 range; (3) baseline voiding dysfunction or abnormal urodynamic studies; (4) active severe urinary tract infection or vesicovaginal fistula; (5) significant impairment of major organ function; and (6) enrollment in another clinical trial within 4 weeks before the start of this study.

### 2.2. Randomization and Blinding

Randomization was performed in a 1:1 allocation ratio, assigning eligible participants to either the control arm (receiving standard bladder training alone) or the TEAS arm (receiving standard bladder training plus transcutaneous electrical acupoint stimulation). The randomization sequence was generated by computer, prepared by an independent biostatistician, and concealed using sequentially numbered, opaque sealed envelopes. Due to the nature of the TEAS intervention, neither patients nor the clinical staff delivering the treatment could be masked to group assignment. Nevertheless, the outcome assessors-who were responsible for measuring residual urine, documenting adverse events, and administering the SF-36 questionnaire-remained blinded to allocation, as did the statistician who performed the final data analysis.

### 2.3. Intervention Protocol

All patients underwent standard radical hysterectomy and received routine postoperative care, including indwelling urinary catheterization. All surgical procedures were nerve-sparing radical hysterectomies (Type C1 according to the Querleu-Morrow classification), performed by the same experienced surgical team to standardize the extent of pelvic autonomic nerve injury. Control group: Patients received standardized bladder training twice daily from postoperative day 3 to day 10. Each session consisted of pelvic floor muscle exercises (Kegel exercises: 5 s contraction followed by 5 s relaxation, repeated for 15 min) and coordinated abdominal breathing (10 deep breaths per cycle, 5 cycles per session). TEAS group: In addition to the identical bladder training regimen, patients received transcutaneous electrical acupoint stimulation (TEAS) once daily for 7 consecutive days, starting on postoperative day 3. TEAS was administered using a low-frequency pulse therapeutic device (HANS-200A, Beijing Jinshan Technology Co., Ltd., Beijing, China). Two self-adhesive surface electrodes (5 cm × 5 cm) were placed on each acupoint. The stimulated acupoints were bilateral BL32 (Ciliao), BL33 (Zhongliao), SP6 (Sanyinjiao), and unilateral CV4 (Guanyuan) and CV3 (Zhongji). Stimulation parameters were set to a dense-disperse wave at 2/100 Hz, with a pulse width of 0.2 ms. The intensity was titrated to a level perceived as strong but not painful (typically 10–25 mA). Each session lasted 30 min.

### 2.4. Outcome Measures

All outcome assessments were performed at baseline (the first day after the operation) and on postoperative days 11 and 30 by assessors blinded to group allocation.

Primary outcome: Urinary retention, defined as post-void residual urine volume (PVR) > 100 mL on postoperative day 11. PVR was measured by bladder ultrasound (Mindray M7, Mindray Medical International Co., Ltd., Shenzhen, China) within 15 min after spontaneous voiding. The average of three measurements was recorded. Secondary outcomes: (1) Post-void residual urine volume (PVR) as a continuous variable. (2) Time to spontaneous voiding recovery: the interval (in hours) from catheter removal to the first spontaneous void with PVR ≤ 100 mL without re-catheterization. (3) Recatheterization: any re-insertion of a urinary catheter within 30 days after initial removal. Indications for recatheterization were post-void residual volume > 100 mL accompanied by significant lower abdominal discomfort, or post-void residual volume > 300 mL regardless of symptoms. All re-insertions were performed as indwelling urethral catheterization. (4) Symptomatic urinary tract infection (UTI): presence of at least one urinary symptom (dysuria, frequency, urgency, suprapubic pain) plus a positive urine culture (≥10^5^ colony-forming units/mL). (5) Treatment efficacy: categorized based on PVR on day 30: cured (PVR < 50 mL), markedly effective (PVR 50–100 mL), effective (PVR 100–200 mL), or ineffective (PVR > 200 mL or inability to void spontaneously). (6) Health-related quality of life: assessed using the 36-Item Short Form Health Survey (SF-36) at baseline and day 30.

### 2.5. Statistical Analysis

Statistical evaluation was carried out with SPSS version 26.0 (IBM Corp., Armonk, NY, USA). Statistical significance was defined as a two-sided *p* value below 0.05. Normality of continuous data was assessed using the Shapiro–Wilk test. Variables following a normal distribution were summarized as mean ± standard deviation and compared between groups using the independent-samples *t*-test. For non-normally distributed variables, data were expressed as median with interquartile range and analyzed with the Mann–Whitney U test. Categorical data were compared using the chi-square test or Fisher’s exact test, as appropriate. The ordinal outcome of treatment efficacy was evaluated with the Mann–Whitney U test. All randomized patients were analyzed according to the intention-to-treat principle. As the follow-up rate reached 100%, no data imputation was required.

### 2.6. Ethical Approval and Consent

The study was conducted in accordance with the Declaration of Helsinki and approved by the Ethics Committee of the Obstetrics and Gynecology Hospital of Fudan University (Approval No. 2023-148) on 26 December 2023. Informed consent was obtained from all subjects involved in the study.

### 2.7. Data Availability

The datasets generated and analyzed during the current study are not publicly available owing to privacy and ethical considerations regarding patient confidentiality. However, de-identified data underlying the statistical analyses reported in this manuscript can be obtained from the corresponding author upon reasonable request, provided that such sharing is approved by the institutional review board and a formal data use agreement is executed. These measures are in place to ensure compliance with applicable ethical standards and institutional policies. This study was reported in accordance with the CONSORT 2025 guidelines [14]. The completed CONSORT checklist is provided as Supplementary Material S1.

### 2.8. Use of Generative AI

No generative artificial intelligence was used for any substantial part of this work, including study design, data collection, analysis, interpretation, or manuscript generation. The use of AI for superficial text editing (e.g., grammar, spelling, punctuation) is not applicable.

## 3. Results

### 3.1. Participant Flow and Baseline Characteristics

**Figure 1:**
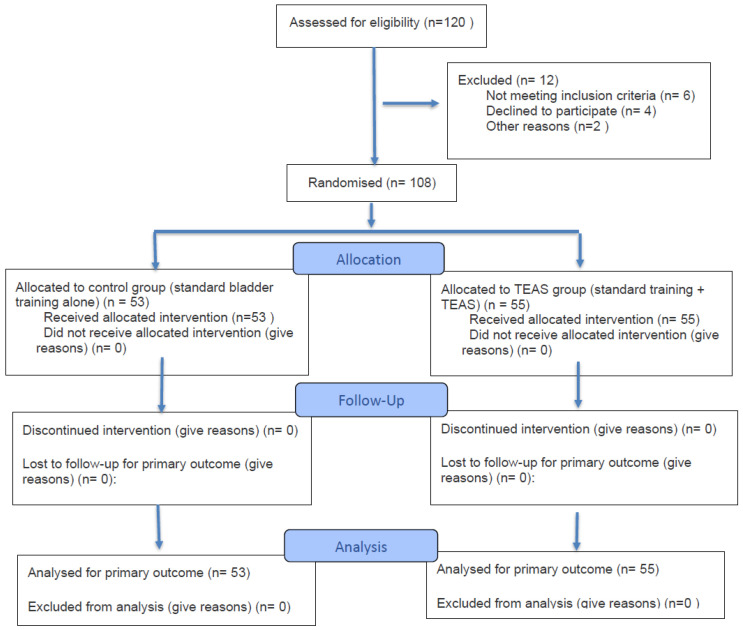
CONSORT flow diagram of the study. A total of 120 patients were assessed for eligibility; 12 were excluded (6 did not meet inclusion criteria, 4 declined to participate, and 2 for other reasons). 108 patients were randomized: 53 to the control group (standard bladder training alone) and 55 to the TEAS group (standard bladder training plus transcutaneous electrical acupoint stimulation). All randomized patients completed the 30-day follow-up and were included in the intention-to-treat analysis.

Between February 2024 and December 2025, 120 patients were assessed for eligibility. Twelve patients were excluded, and 108 were randomized: 53 to the control group (standard bladder training alone) and 55 to the TEAS group (standard bladder training plus transcutaneous electrical acupoint stimulation). All randomized patients completed the study and were included in the intention-to-treat analysis (Figure 1, CONSORT flow diagram).

Comparisons between the two groups revealed no statistically significant differences in any baseline demographic or clinical variables, including age, body mass index, parity, diabetes, pathology type, FIGO stage, operative time, or estimated blood loss (all *p* > 0.05; Table 1).

### 3.2. Primary Outcome

As shown in Table 2, the incidence of urinary retention (PVR > 100 mL on postoperative day 11) was 35.8% (19/53) in the control group and 23.6% (13/55) in the TEAS group. The difference was not statistically significant (χ^2^ = 1.94, *p* = 0.164).

### 3.3. Secondary Outcomes

The TEAS group showed significantly better results across multiple secondary bladder-emptying measures (Table 2). Post-void residual urine volume (PVR): The median PVR was significantly lower in the TEAS group (70 mL [IQR 40–90]) than in the control group (85 mL [IQR 45–145]; Mann–Whitney U = 1032.5, *p* = 0.004). For descriptive purposes, the mean ± SD was 90.3 ± 66.3 mL in the TEAS group and 122.4 ± 72.7 mL in the control group. The median time to spontaneous voiding recovery was significantly shorter in the TEAS group (2 h [IQR 1–4]) than in the control group (4 h [IQR 2–13]; Mann–Whitney U = 987.0, *p* < 0.001). The mean ± SD was 5.9 ± 7.2 h in the TEAS group and 12.6 ± 14.8 h in the control group. The recatheterization rate was significantly lower in the TEAS group (16.4%, 9/55) than in the control group (34.0%, 18/53; χ^2^ = 4.40, *p* = 0.036). The incidence of Urinary tract infection (UTI) was 18.2% (10/55) in the TEAS group and 24.5% (13/53) in the control group. This difference was not statistically significant (χ^2^ = 0.65, *p* = 0.421). The distribution of treatment efficacy (cured/markedly effective/effective/ineffective) differed significantly between groups, favoring the TEAS group (Mann–Whitney U, *p* = 0.009; Table 2). The proportion of patients classified as “cured” was 36.4% (20/55) in the TEAS group versus 9.4% (5/53) in the control group.

### 3.4. Quality of Life Outcomes

At baseline, there were no significant between-group differences in any of the eight SF-36 domains (Physical Functioning [PF], Role Physical [RP], Bodily Pain [BP], General Health [GH], Vitality [VT], Social Functioning [SF], Role Emotional [RE], and Mental Health [MH]; all *p* > 0.05). Significant within-group improvements from baseline to day 30 were observed for all domains in both groups (all *p* < 0.001). At day 30, the TEAS group scored significantly higher than the control group across all eight dimensions (all *p* < 0.05), with significantly greater improvements from baseline in the TEAS group for every domain (all *p* < 0.05).

### 3.5. Adverse Events

No serious adverse events related to TEAS were observed. Mild local skin discomfort was reported in 2 patients (3.6%) in the TEAS group, which resolved spontaneously without intervention. No adverse events were reported in the control group.

## 4. Discussion

This randomized controlled trial provides evidence that adjunctive transcutaneous electrical acupoint stimulation (TEAS) improves several clinically important outcomes after radical hysterectomy for cervical cancer. Although TEAS did not significantly reduce the primary outcome of urinary retention, it significantly lowered the recatheterization rate, reduced post-void residual urine volume, shortened the time to spontaneous voiding recovery, enhanced treatment efficacy, and substantially improved quality of life. These consistent favorable effects across multiple objective measures suggest that TEAS provides meaningful clinical benefit for postoperative bladder rehabilitation.

Our findings align with and extend previous evidence supporting neuromodulatory interventions for postoperative voiding dysfunction [6,9,10]. The significant reduction in residual urine volume and accelerated return of spontaneous voiding suggest that TEAS actively facilitates neurological recovery rather than providing merely symptomatic relief. These findings are consistent with a recent meta-analysis of 22 RCTs (*n* = 1563), which demonstrated that acupuncture significantly improved treatment response and reduced urinary tract infections in patients with urinary retention after radical hysterectomy for cervical cancer [15]. While that evidence was derived primarily from acupuncture and electroacupuncture studies, our findings further extend this evidence by confirming that TEAS, as a less invasive alternative, provides comparable benefits. A recent systematic review by Liu and Chien [16] demonstrated that electrical stimulation improved voiding outcomes after anorectal surgery, but data specifically for gynecologic oncology remained limited. Our study fills this gap by providing evidence from a well-conducted RCT in cervical cancer patients undergoing radical hysterectomy. The observed improvement in the recatheterization rate is clinically meaningful, as recatheterization is burdensome for patients and increases healthcare costs and infection risk. The concept of multimodal prehabilitation has gained attention in gynecologic oncology, with a recent review by Kim et al. [16] reporting that programs combining physical exercise, nutritional support, and psychological intervention can shorten hospital stay, reduce complications and transfusion rates, and accelerate gastrointestinal recovery. Our findings extend this concept in two ways. First, TEAS achieved comparable improvements in bladder function—reducing recatheterization from 34.0% to 16.4% and shortening voiding recovery from 4 h to 2 h—using a much shorter intervention (7 days, starting on postoperative day 3) than typical prehabilitation programs (2–4 weeks preoperatively). Second, while psychological interventions in prehabilitation have shown inconsistent effects [17], our TEAS intervention produced consistent improvements across all SF-36 domains, including mental health and emotional well-being, possibly by relieving the distress associated with voiding dysfunction and catheter dependence.

The robust improvement in patient-reported quality of life is particularly noteworthy. The mean increases in SF-36 exceeded the important difference reported for this instrument [18]. At 30 days postoperatively, the TEAS group achieved significantly higher scores than the control group in all eight SF-36 domains (all between-group *p* < 0.05). Moreover, the improvement from baseline to day 30 (Δ) was significantly greater in the TEAS group than in the control group for every domain, as reflected by the Δ between-group *p* values ranging from 0.001 to 0.047 (Table 3). These findings indicate that the benefit of TEAS extends beyond resolving a specific complication, positively impacting multiple dimensions of daily living and emotional well-being. By mitigating bladder dysfunction, TEAS likely interrupts the detrimental cycle of voiding difficulty, restricted physical activity, and psychological distress, thereby supporting holistic postoperative recovery-a goal fully aligned with enhanced recovery after surgery (ERAS) protocols.

The neurophysiological rationale for TEAS is compelling. Low-frequency electrical stimulation applied to sacral and lower abdominal acupoints (BL32, BL33, CV3, CV4) is believed to modulate the sacral micturition center (S2–S4), potentially re-coordinating detrusor and sphincter function [19]. The selected acupoints have distinct anatomical relevance to bladder innervation. BL32 and BL33 overlie the sacral foramina, near the sacral nerve roots (S2–S4) that provide parasympathetic innervation to the detrusor, while CV3 and CV4 are midline points on the lower abdomen traditionally associated with pelvic floor and bladder function [19]. Beyond this anatomical rationale, a recent study by Tu et al. [20] found that TEAS at these points significantly increased urinary ATP levels in elderly patients after hip arthroplasty, suggesting a metabolic pathway through which electrical stimulation may facilitate detrusor contraction and improve voiding efficiency. This observation offers a plausible molecular explanation for the reduced residual volumes and faster recovery seen in our TEAS group. In addition, improved local pelvic microcirculation may reduce postoperative edema, creating a more favorable environment for nerve repair [16,21]. The early initiation of TEAS (postoperative day 3) may be critical, as it precedes the peak of postoperative inflammatory edema. These mechanistic hypotheses are supported by the significantly better outcomes observed in the TEAS group across multiple bladder-emptying measures. The analgesic and neuromodulatory effects of electrical stimulation have also been demonstrated in other surgical settings, where percutaneous electrical nerve stimulation provided comparable benefits to acupuncture for postoperative pain control [22].

Several limitations should be acknowledged. First, this study is part of an ongoing four-arm trial; the present findings are derived from the comparison between the control and TEAS groups, which completed enrollment and follow-up ahead of the other two arms due to faster recruitment. The remaining two arms are still recruiting, and the full four-arm results will be reported separately. Therefore, the present findings should be interpreted as interim in nature. Second, the single-center design may limit generalizability; multicenter validation is needed. Third, the open-label nature of the intervention introduces potential performance bias, although outcome assessors and data analysts were blinded. Fourth, the study was not powered for the observed difference in urinary retention; the sample size provides limited statistical power for the primary analysis. Fifth, the follow-up period was limited to 30 days. While this timeframe captures the critical early recovery phase, we acknowledge that bladder function recovery after radical hysterectomy can take up to one year, and our current data cannot confirm whether the early benefits of TEAS are sustained long-term. We are continuing to follow this cohort and plan to report 12-month outcomes separately. Sixth, TEAS parameters (frequency, duration, timing) were fixed; optimization studies are warranted. Seventh, the inclusion criteria restricted the patient age to 35–60 years. While this was intended to reduce confounding from hormonal variations in younger patients and comorbidities in older patients, it inevitably limits the generalizability of our findings to the broader cervical cancer population, particularly to those at the extremes of age. Consequently, whether TEAS offers comparable benefits for patients under 35 or over 60 years remains to be determined.

Future research should focus on larger, multi-center, adequately powered RCTs to confirm the effects of TEAS on urinary retention and patient-important outcomes. Optimization of stimulation parameters (e.g., different frequencies, treatment durations, or starting times) may further enhance efficacy. Additionally, mechanistic studies using urodynamic or neurophysiological measurements could clarify how TEAS modulates bladder function at the neural level. The significant improvement in quality of life observed in our study also suggests that future trials should incorporate patient-reported outcome measures as primary or co-primary endpoints, as these may better reflect the holistic benefits of TEAS beyond objective urodynamic parameters. From a clinical implementation perspective, cost-effectiveness analyses would help determine the value of integrating TEAS into routine enhanced recovery protocols. Given the relatively low cost of TEAS devices and the minimal training required for their operation, TEAS could be a scalable intervention in both high-resource and low-resource settings, provided that its efficacy is confirmed in larger trials.

## 5. Conclusions

In this randomized controlled trial, adjunctive transcutaneous electrical acupoint stimulation (TEAS) did not significantly reduce the primary outcome of urinary retention after radical hysterectomy for cervical cancer. However, it significantly improved multiple secondary outcomes, including recatheterization rate, residual urine volume, recovery time, treatment efficacy, and quality of life. TEAS is a safe, non-invasive, well-tolerated therapy that deserves consideration for integration into enhanced recovery protocols. Larger, definitive RCTs are warranted to confirm these findings.

## Figures and Tables

**Table 1:** Baseline demographic and clinical characteristics of participants.

Characteristic	Control Group (*n* = 53)	TEAS Group (*n* = 55)	Statistical Test	*p*
Age (years) Mean ± SD	48.7 ± 5.8	47.9 ± 5.4	t = 0.741	0.432
BMI (kg/m^2^) Mean ± SD	23.5 ± 2.3	22.9 ± 2.1	t = 1.414	0.157
Parity, *n* (%)			χ^2^ = 0.06	0.891
-0	5 (9.4)	6 (10.9)		
-≥1	48 (90.6)	49 (89.1)		
Diabetes, *n* (%)			Fisher’s exact	0.812
-YES	6 (11.3)	7 (12.7)		
-NO	47 (88.7)	48 (87.3)		
Pathological *n* (%)			χ^2^ = 0.01	0.943
-SCC	37 (69.8)	38 (69.1)		
-Adenocarcinoma	16 (30.2)	17 (30.9)		
FIGO Stage *n* (%)			Fisher’s exact	0.876
-IB1	11 (20.8)	10 (18.2)		
-IB2	13 (24.5)	15 (27.3)		
-IB3	10 (18.9)	9 (16.4)		
-IIA1	12 (22.6)	14 (25.5)		
-IIA2	7 (13.2)	7 (12.7)		
Operative Time (min), Mean ± SD	141.3 ± 15.2	138.6 ± 14.7	t = 0.938	0.341
Blood Loss (mL), Mean ± SD	208.5 ± 45.3	202.7 ± 43.8	t = 0.676	0.498

TEAS = transcutaneous electrical acupoint stimulation; BMI = body mass index; SD = standard deviation; FIGO = International Federation of Gynecology and Obstetrics. Normally distributed continuous variables were compared using independent samples *t*-test. Categorical variables were compared using χ^2^ test or Fisher’s exact test (when expected frequency < 5).

**Table 2:** Primary and secondary outcomes.

Outcome Variable	Control Group (*n* = 53)	TEAS Group (*n* = 55)	Statistical Test	*p*
Primary outcome				
Urinary retention (PVR > 100 mL), *n* (%)	19 (35.8)	13 (23.6)	χ^2^ = 1.94	0.164
Secondary outcomes				
Post-void residual urine volume (mL)				
Median (IQR)	85 (45–145)	70 (40–90)	U = 1032.5	0.004
Mean ± SD	122.4 ± 72.7	90.3 ± 66.3	descriptive	
Time to spontaneous voiding (hours)				
Median (IQR)	4 (2–13)	2 (1–4)	U = 987.0	<0.001
Mean ± SD	12.6 ± 14.8	5.9 ± 7.2	descriptive	
Recatheterization, *n* (%)	18 (34)	9 (16.4)	χ^2^ = 4.40	0.036
Urinary tract infection, *n* (%)	13 (24.5)	10 (18.2)	χ^2^ = 0.65	0.421
Treatment efficacy, *n* (%)			U	0.009
Cured	5 (9.4)	20 (36.4)		
Markedly effective	29 (54.7)	22 (40)		
Effective	11 (20.8)	7 (12.7)		
Ineffective	8 (15.1)	6 (10.9)		

TEAS = transcutaneous electrical acupoint stimulation; PVR = post-void residual urine volume; IQR = interquartile range; SD = standard deviation. For non-normally distributed continuous variables (residual urine, recovery time) and the ordinal variable (treatment efficacy), Mann–Whitney U test was used. For categorical variables, χ^2^ test was used. Bold *p* values indicate statistical significance (*p* < 0.05). Mean ± SD values are provided for descriptive purposes only and were not used for between-group comparisons.

**Table 3:** Comparison of SF-36 domain scores between Control and TEAS groups.

Domain	Group	Baseline (Mean ± SD)	30-Day (Mean ± SD)	Within-Group *p* ^1^	Baseline Between-Group *p*	30-Day Between-Group *p*	Δ Between-Group *p* ^2^
PF	Control	55.0 ± 6.3	57.2 ± 6.5	<0.001	0.231	<0.001	<0.001
	TEAS	56.4 ± 6.1	61.9 ± 4.9	<0.001			
RP	Control	58.1 ± 4.7	63.5 ± 5.1	<0.001	0.782	<0.001	0.003
	TEAS	58.4 ± 5.5	66.8 ± 4.2	<0.001			
BP	Control	58.3 ± 5.0	67.8 ± 4.3	<0.001	0.792	<0.001	0.001
	TEAS	58.6 ± 6.0	71.3 ± 3.8	<0.001			
GH	Control	62.4 ± 5.1	70.1 ± 4.8	<0.001	0.119	0.048	0.002
	TEAS	60.8 ± 5.3	71.6 ± 4.6	<0.001			
VT	Control	57.2 ± 5.8	61.9 ± 5.7	<0.001	0.135	<0.001	0.001
	TEAS	58.9 ± 5.9	66.9 ± 5.0	<0.001			
SF	Control	59.0 ± 4.8	65.5 ± 5.4	<0.001	0.853	0.002	0.007
	TEAS	59.2 ± 5.2	68.4 ± 4.7	<0.001			
RE	Control	57.9 ± 5.5	68.7 ± 5.0	<0.001	0.690	0.01	0.047
	TEAS	58.3 ± 5.4	71.1 ± 4.3	<0.001			
MH	Control	59.3 ± 5.6	68.6 ± 5.2	<0.001	0.851	0.002	0.013
	TEAS	59.5 ± 5.7	71.5 ± 4.5	<0.001			

^1^ Within-group *p* values compare baseline vs. day 30 within each group (paired *t*-test or Wilcoxon signed-rank test). ^2^ Δ Between-group *p* values compare the change from baseline to day 30 (day 30 minus baseline) between the control and TEAS groups (independent *t*-test or Mann–Whitney U test). No significant between-group differences were observed at baseline for any domain (all *p* > 0.05). TEAS = transcutaneous electrical acupoint stimulation; SD = standard deviation.

## Data Availability

The data supporting the findings of this study are available from the corresponding author upon reasonable request. Due to privacy and ethical restrictions related to patient confidentiality, the raw data are not publicly archived. De-identified datasets used for the statistical analyses presented in this manuscript may be shared with qualified researchers for purposes of reproducing or verifying the results, subject to institutional review board approval and a signed data use agreement.

## Supplementary Materials

The following supporting information can be downloaded at: https://www.mdpi.com/article/10.3390/curroncol33070424/s1, Supplementary Materials S1: The CONSORT 2025 Statement is cited in the main text as reference [14].

## Abbreviations

The following abbreviations are used in this manuscript:

TEAS	transcutaneous electrical acupoint stimulation
PVR	post-void residual urine volume
UTI	urinary tract infection
SF-36	36-Item Short Form Health Survey
IQR	interquartile range
SD	standard deviation
RCT	randomized controlled trial
ERAS	enhanced recovery after surgery
BMI	body mass index
SCC	squamous cell carcinoma
FIGO	International Federation of Gynecology and Obstetrics
